# Poorly differentiated thyroid carcinoma: An underdiagnosed entity

**DOI:** 10.1007/s00292-019-0600-9

**Published:** 2019-07-04

**Authors:** M. S. Dettmer, A. Schmitt, P. Komminoth, A. Perren

**Affiliations:** 1 Institute of Pathology, University of Bern, Murtenstraße 31, 3010 Bern, Switzerland; 2 Institute of Pathology, City Hospital Triemli, 8063 Zürich, Switzerland

**Keywords:** Poorly differentiated thyroid carcinoma, MicroRNA, Prognosis, Thyroid neoplasm, Tyrosine kinase inhibitor, Gering differenziertes Schilddrüsenkarzinom, MicroRNA, Prognose, Schilddrüsentumor, Thyrosinkinaseinhibitor

## Abstract

Poorly differentiated thyroid carcinomas (PDTCs) are a rare subtype of thyroid carcinomas that are biologically situated between well-differentiated papillary/follicular thyroid carcinomas and anaplastic thyroid carcinomas (ATCs).

The diagnosis of conventional as well as oncocytic poorly differentiated thyroid carcinoma is difficult and often missed in daily routine. The current WHO criteria to allow the diagnosis of PDTCs are based on the results of a consensus meeting held in Turin in 2006. Even a minor poorly differentiated component of only 10%of a given carcinoma significantly affects patient prognosis and the oncocytic subtype may even have a worse outcome. Immunohistochemistry is not much help and is mostly used to exclude a medullary thyroid carcinoma with calcitonin and to establish a follicular cell of origin via thyroglobulin staining.

Due to the concept of stepwise dedifferentiation, there is a vast overlap of different molecular alterations like *BRAF, RAS, CTNNB1, TP53* and others between different thyroid carcinoma subtypes. A distinctive molecular tumor profile is therefore currently not available.

PDTCs have a unique miRNA signature, which separates them from other thyroid carcinomas. The average relapse free survival is less than one year and about 50% of patients die of the disease. Modern tyrosine kinase inhibitors offer in conjunction with powerful molecular diagnostic new chances in these difficult to treat carcinomas.

## Abstract (de)

Gering differenzierte Schilddrüsenkarzinome (PDTC) stellen einen seltenen Subtyp des Schilddrüsenkarzinoms dar, welcher biologisch zwischen den gut differenzierten Schilddrüsenkarzinomen (papilläre und follikuläre Schilddrüsenkarzinome) auf der einen Seite und den anaplastischen Schilddrüsenkarzinomen auf der anderen Seite angesiedelt ist.

Die Diagnose des konventionellen wie des onkozytären PDTC ist schwierig und wird in der täglichen Routine oft verpasst. Derzeit werden diese Tumoren nach Kriterien der aktuelle WHO-Klassifikation diagnostiziert, welche in einer Konsensustagung 2006 in Turin erarbeitet wurden. Selbst ein kleiner Anteil eines PDTC von nur 10 % innerhalb eines Schilddrüsenkarzinoms beeinflusst die Prognose nachhaltig und die onkozytäre Variante hat einen nochmals ungünstigeren Verlauf. Immunhistochemische Analysen sind meist nicht hilfreich und werden genutzt, um ein medulläres Schilddrüsenkarzinom mittels Calcitonin auszuschließen oder die Follikelepithelzelle als Ursprungszelle mittels Thyreoglobulin nachzuweisen.

Auf molekularer Ebene gibt es eine große Überlappung unterschiedlicher Mutationen in den verschiedenen Schilddrüsenkarzinomen wie *BRAF*, *RAS*, *CTNNB1* oder *TP53*, welche sich durch das Konzept der schrittweisen Dedifferenzierung gut erklären lassen. Entsprechend existiert nach wie vor kein eigenständiges, für die Diagnostik einsetzbares molekulares Profil. PDTC haben ein distinktes miRNA-Profil im Vergleich zu anderen Schilddrüsenkarzinomen. Das durchschnittliche rezidivfreie Überleben liegt unter einem Jahr und etwa 50 % der Patienten versterben an ihrem Tumor. Thyrosinkinaseinhibitoren eröffnen gemeinsam mit einer leistungsstarken molekularen Diagnostik neue Therapiechancen in diesen schwer zu therapierenden Karzinomen.

## Background

Theodor Langhans described a malignant thyroid carcinoma in 1907, which he termed “rampantly goiter” or “wuchernde Struma” in German [30]. While this entity was well received in the German literature and textbooks [41], the term “poorly differentiated thyroid carcinoma” (PDTC) was first introduced by Granner and Buckwalter in the early 1960s to the English-speaking audience. At that time, clear diagnostic criteria were not provided [21].

With pathology developing a more criteria-based approach, two groups independently published different diagnostic criteria in 1983/1984 to allow for the diagnosis of PDTC [12, 44]. Sakamoto et al. postulated the frequency of PDTC at about 14% in a series of 258 thyroid carcinomas [44]. The other group referred to Langhans’ so-called wuchernde Struma, which he introduced 1907 and which may in fact be the first description of a PDTC in the literature [12].

The two schools of thought remained: While one school placed more emphasis on the growth pattern of the lesion (trabecular, insular, or solid), the other group used typical features of high-grade lesions like atypia, tumor necrosis, or a high mitotic index [1, 12, 38, 44, 49, 54]. It took another 20 years for this entity to be accepted and introduced in the World Health Organization (WHO) series of malignant human neoplasms in 2004 [13] and since then it has been a recognized entity [33].

Both of the aforementioned groups recognized the concept of a thyroid tumor that is placed biologically between well-differentiated thyroid carcinomas like papillary thyroid carcinomas (PTC) or follicular thyroid carcinomas (FTC) with an excellent prognosis and the extremely aggressive anaplastic thyroid carcinomas (ATC; [36]). However, both groups offered significant different diagnostic approaches to allow for such a diagnosis. Obviously, different diagnostic algorithms and criteria led to an increased uncertainty of these lesions and, consequently, studies were not comparable. While some integrated tumors that we would classify as PDTC today, others also included the tall cell variant of PTC, which today would fall into the category of well-differentiated thyroid carcinomas (however, with an adverse outcome; [27, 45]).

## Diagnostic algorithm

**Fig. 1:**
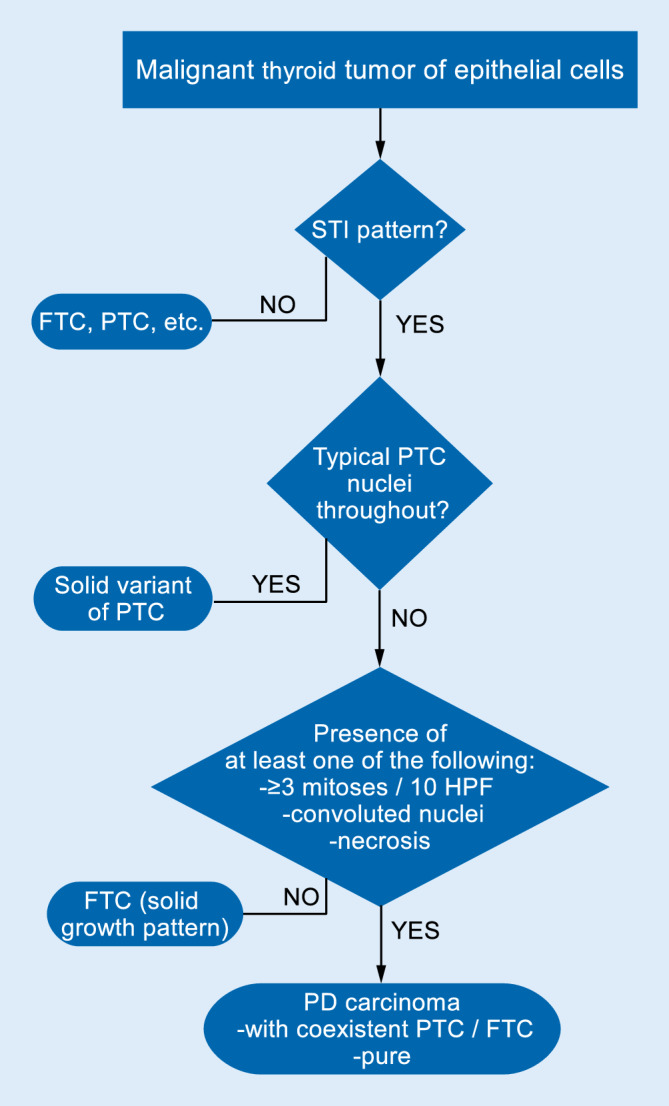
Diagnostic algorithm for the diagnosis of a poorly differentiated thyroid carcinoma (*PDTC*). *FTC* follicular thyroid carcinomas, *PTC* papillary thyroid carcinomas, *STI* solid, trabecular, or insular growth, *PD* poorly differentiated, *HPF* high power field

**Fig. 2:**
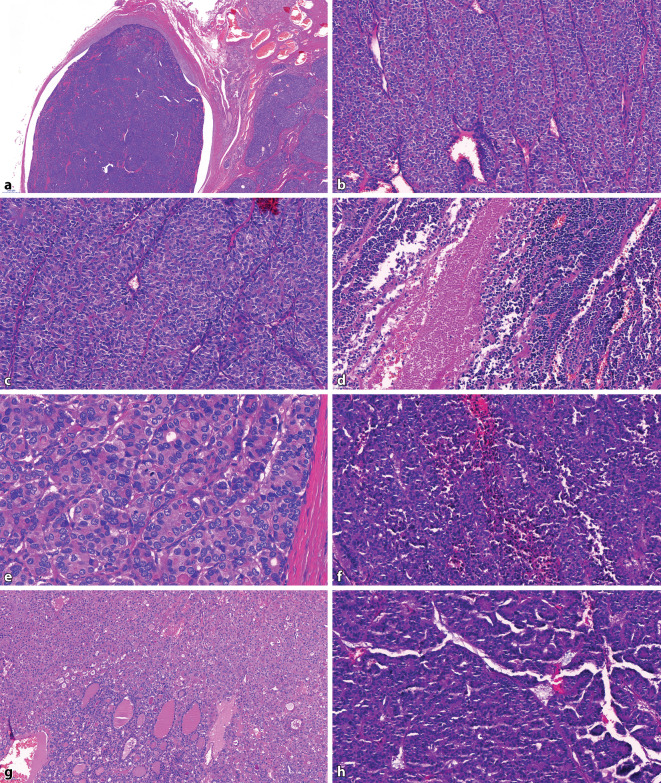
**a** Obviously malignant neoplasm, vascular invasion and invasion into adjacent tissue; **b** trabecular growth pattern; **c** insular and solid growth pattern; **d** tumor necrosis; **e** mitosis and psammoma bodies, no papillary thyroid carcinomas (PTC) nuclei; **f** raisinoid nuclei, trabecular growth pattern; **g** *upper part* oncocytic poorly differentiated thyroid carcinoma (PDTC), *lower part* FTC; **h** PTC remnants in a PDTC. Only architecture of PTC, no longer any PTC nuclei

In order to unify the concepts of PDTC, a group of internationally recognized thyroid experts met in 2006 in Turin and elaborated the so-called Turin criteria, which are a diagnostic algorithm, based on both schools of thought: high-grade features and growth pattern (Fig. 1 and 2; [53]). This algorithm is now accepted and integrated in the WHO classification [33].

After identification of malignancy (angioinvasion and/or gross invasion), a so-called STI pattern (solid, trabecular, or insular growth pattern) is the first hint of the diagnosis of PDTC. It is worth pointing out that PDTC may on rare occasions be completely encapsulated and even without angioinvasion. These cases are prone to be misdiagnosed as adenomas if the pathologist in charge is not familiar with the concept of PDTC.

One has to look for PTC nuclei throughout the lesion in order to exclude the solid variant of PTC. If this feature is absent, one of the three following features is enough to allow for the diagnosis of a PDTC to be made:

1. convoluted nuclei that are a bit smaller than those of a PTC while also having a wrinkled irregular contour that overall gives them a raisinoid-like appearance. Nevertheless, their chromatin is much darker and evenly distributed and their overall appearance is more uniform than in PTC nuclei. Nuclear pseudoinclusions are absent while one may observe some nuclear grooves. If pseudoinclusions are present, they may be seen as a sign of tumor progression from a PTC to a PDTC, and if enough tumor material is embedded, classic PTC remnants may be seen in such cases [36].
2. Tumor necrosis also allows for the diagnosis of a PDTC in this flowchart, usually seen as a small necrotic focus within a solid tumor cell nest. Single cell necrosis does not count, and it is essential to exclude tumor necrosis followed by fine-needle aspiration (FNA), which happens frequently especially in oncocytic nodules.
3. The last criterion is an increased mitotic activity of at least three mitoses per ten high-power fields. This cut-off exhibited a good correlation with survival data in the cases used in the Turin consensus meeting; however, the amount of mitosis per mm^2^ needed was unfortunately not defined [54].

Applying the correct Turin criteria, PDTCs are—as already indicated in the title—rare lesions. Over a time span of 26 years and in a population of 1.37 million inhabitants, we were able to identify 34 PDTC carcinomas [15]. In that study, only one tumor was diagnosed as PDTC in the original pathology report, indicating that the concept of PDTC is not yet well known and must be used more widely in everyday practice [14].

Currently, the incidence of PDTC in Europe is estimated at 4–7% of all thyroid carcinomas, which corresponds to 240 new cases in Germany each year.

The incidence of indolent thyroid carcinomas has been rising for many years and as a consequence many patients get overtreated [34]. On the other hand, PDTCs, which require a correct diagnosis in order to receive adequate treatment, are often missed in daily routine.

## Fine-needle aspiration

The cytological diagnosis of PDTC on FNA samples is challenging. This is due to the rareness of the diagnosis, the nonspecific cytological features, the overlap with cytological characteristic of follicular neoplasms, and the frequently encountered sampling error of the PD component in an otherwise well-differentiated tumor. Thus, according to published series, only 27% of cases were diagnosed correctly as PDTC on FNA [3, 5, 28, 40], whereas most of the remaining cases were put into the category “(suggestive of) follicular neoplasm”[6]. According to a recent review and meta-analysis of the literature by Saglietti et al., the presence of insular or solid architecture, hypercellularity, high nuclear/cytoplasmic ratio, and mitotic activity can suggest the diagnosis of PDTC [43].

## How much “poorly differentiated” is needed?

**Fig. 3:**
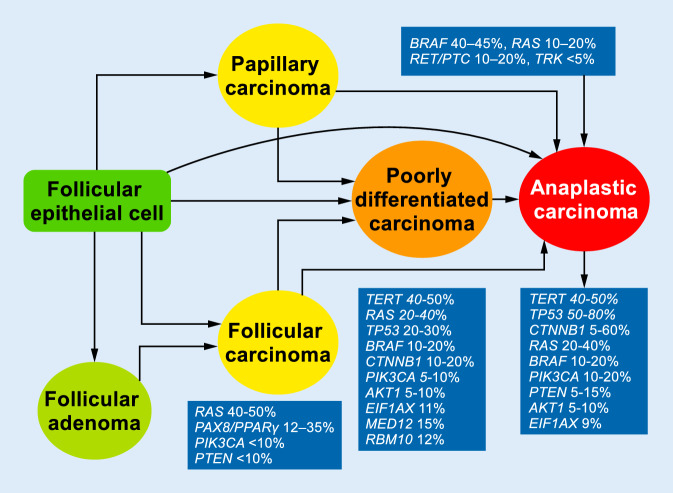
Stepwise de-differentiation of thyroid carcinomas derived from follicular epithelial cell

It has long been known that there are PDTCs with a minor and even a major component of a well-differentiated thyroid carcinoma like PTC or FTC. Today, it is believed that most of them arise in a well-differentiated thyroid carcinoma, although a subset of these lesions apparently also arise de novo (Fig. 3). About 80% of PDTC have a PD component of >50%, and only 20% of PDTC have a minor PD component [14]. However, it was unclear in the Turin proposal what percentage of poorly differentiated was needed in a tumor to allow for such a diagnosis and—more importantly—to affect patient prognosis; moreover, the 2004 WHO classification did not offer a cut-off value [13]. We showed that even a small PD component of 10% affects patient prognosis equally negative as a tumor that consists of 100% PD regarding relapse-free survival or overall survival [14, 33]. Our findings were recently confirmed by others [4] and are now reflected in the current WHO classification [33]. Therefore, a thorough histological work-up is highly recommended to ensure an adequate diagnosis is made.

## Oncocytic subtype—does it matter?

Based on their neoplastic tumor of origin, PDTCs can be subdivided into papillary type, follicular type, and not otherwise specified (NOS). However, this subdivision does not have any clinical or prognostic consequences [36].

Owing to a lack of data, oncocytic carcinomas were excluded in the Turin proposal and therefore it was initially unknown whether these criteria were also applicable to oncocytic lesions. We reported that the Turin criteria can also be applied in this scenario [15]. However, it should be emphasized that the presence of necrosis in these tumors should not be overestimated since oncocytic lesions in general are prone to spontaneous or FNA-initiated infarction and focal necrosis. Thus, one should carefully look for signs of previous FNA in a given case. And while the relapse-free survival is similar between PDTC and oncocytic PDTC, it is nevertheless very important to distinguish between PDTC and oncocytic PDTC, since oncocytic PDTC may have an even worse overall survival than conventional PDTC [15].

## Differential diagnoses

Poorly differentiated thyroid carcinoma must be distinguished from malignant lesions that are not derived from follicular epithelial cells such as medullary thyroid carcinomas or rarely metastases to the thyroid, most often derived from the kidney or lung [36]. Also, well-differentiated thyroid carcinomas like PTC and especially the solid variant of PTC needs to be excluded on the basis of nuclear features. The same is true for FTCs that do not show an STI pattern. Of note, many lesions that have been classified as widely invasive FTC in the past, today fall into the category of PDTC. And while there is a progression from well-differentiated thyroid carcinomas to PDTC, there may also be a further progression to an ATC that of course is prognostically relevant and even the smallest ATC component should be reported as well [50, 51].

## TNM classification

The current 8th edition of the TNM classification was introduced in 2017. Now, all thyroid carcinomas including ATC are similarly categorized into different tumor stages [9]. One study explored the validity of the new classification and while it seems that a good prognostic separation for FTC can be achieved, neither the 7th nor the 8th edition is able to predict accurately patient prognosis for PDTC [26].

## Immunohistochemistry

Immunohistochemical studies in thyroid carcinomas in general and in PDTC in particular have been performed [31] and results are summarized in Table 1. It can be of help to establish a follicular cell of origin and exclude a medullary thyroid carcinoma, which would stain positive for calcitonin [20] and neuroendocrine markers [31]. Poorly differentiated thyroid carcinomas are typically positive for thyroglobulin, although the expression is often patchy and focal [2, 37]. Strong and diffuse positivity of thyroglobulin is typically found in the adjacent well-differentiated carcinoma component.

**Table 1:** Immunohistochemistry in different thyroid carcinomas

Immunohistochemical staining	MTC	PTC	FTC	PDTC	ATC
Calcitonin	++	−	−	−	−
Chromogranin A	++	−	−	no data	−
Synaptophysin	++	−/+	−/+	no data	−
Thyroglobulin	−	+++	+++	−/+	−/+
Galectin-3	−/+	++	−/+	−/+	−/+
HBME-1	−/+	++	−/+	−/+	−/+
PanCK	++	++	++	+	−/+
TTF1	++	++	++	−/+	−
CK7	++	++	++	−/+	−/+
CK19	−/+	++	−/+	−/+	−
PAX8	−	++	−/+	−/+	−/+

*MTC* medullary thyroid carcinoma, *PTC* papillary thyroid carcinoma, *FTC* follicular thyroid carcinoma, *PDTC* poorly differentiated thyroid carcinoma, *ATC* anaplastic thyroid carcinoma

Most of the diagnostic work-up, however, is based on classic hematoxylin–eosin staining. Markers of malignancy in thyroid carcinomas like Galectin-3 or HBME-1 have been explored, but there is no practical application because signs of malignancy need to be present unequivocally in the first step to enter the Turin criteria [2, 31, 39, 54]. Thus, CD31 staining or similar vascular markers to substantiate angioinvasion are probably more helpful than the aforementioned other antibodies.

About 40–70% of PDTC express TP53—which does not always correlate with the mutational *TP53* gene status—and an immunohistochemical loss of the tumor suppressors p21 and p27 has also been observed [2, 31, 36].

## Molecular alterations

*BRAF* mutations are seen in PDTC in about 20% of cases and *RAS* mutations in 20–40%. Both mutations can, however, also be found in PTC and FTC ([35]; Fig. 3). The activation of MAPK and PI3K–AKT signaling pathways is important for thyroid cancer initiation and progression. Consistent with this notion, mutations in *PIK3CA* can be found in a subset of FTC (10%) and in PDTC cases (5–10%; [36]).

Mutations in the tumor suppressor gene *TP53* and *EIF1AX* are thought to represent a late event in thyroid tumorigenesis and can be detected in about 30% and 11% of PDTC cases [29, 35]. These mutations occur basically only in advanced thyroid carcinomas (PDTC and ATC) and are almost never found in well-differentiated lesions like PTC or FTC [11, 35]. The other mutation that is a putative late event in thyroid cancer progression is *CTNNB1,* which is never found in well-differentiated cancers but in PDTC in about 10–20% and in ATC in up to 60% of cases. Mutations in *MED12* and *RBM10* are recently described mutations in PDTC occurring in 12–15% of cases and may represent novel markers of aggressiveness [23].

Telomerase reverse transcriptase (*TERT*) promoter mutations have been reported in various thyroid malignancies and are always associated with an adverse outcome, probably due to an increased telomerase activity [19, 32]. They occur in two exclusive hotspots, C228T and C250T, with a frequency of 33.8% and 15%, respectively, in PDTC, according to a meta-analysis [32].

Rearrangements are found in 10–14% of PDTCs. These include rearrangements of *RET/PTC, BRAF, ALK, NTRK3,* and *PAX8-PPRγ* [57], which can be encountered in about 7% of cases according to one study [7], while *RET/PTC *rearrangements also seem to be—if at all—only evident in a small percentage of cases [48]. These alterations are more frequently found in well-differentiated tumors—*PAX8-PPRγ* in FTC in about 12–30% and *RET-PTC* in PTC in 10–20% of cases [7, 36]. It is possible, but yet to be proven, that these clones are less aggressive and usually become outgrown by more aggressive tumor parts with other mutations and are therefore rarely found in PDTC.

Owing to the vast overlap of mutations in the different subtypes and the lack of specific mutations, molecular testing of PDTC as a diagnostic tool has not found its way into clinical practice so far. However, the deeper insight into the molecular alterations and the new tyrosine kinase inhibitors will most likely change this in the very near future to ensure adequate patient treatment.

Comparative genomic hybridization (CGH) shows large numbers of chromosomal abnormalities in thyroid cancers. Gains of 5p15, 5q11–13, 19p, and 19q and loss of 8p suggest that these tumors have a common signaling pathway and origin, while gains of 1p34–36, 6p21, 9q34, 17q25, and 20q and losses of 1p11-p31, 2q32–33, 4q11–13, 6q21, and 13q21–31 most likely represent secondary events of progression as they are only found in PDTC and ATC. Finally, gains at 3p13–14 and 11q13 and loss of 5q11–31 are only found in ATC [56]. In short, the number of gains and losses in PDTC are in the range between well-differentiated thyroid carcinomas and ATC, which have—unsurprisingly—the most alterations [42, 56].

## Epigenetic changes

MiRNA expression in poorly differentiated carcinomas has been explored and we know today that PDTCs have a miRNA profile that is not only distinctive from well-differentiated thyroid carcinomas but that also separates PDTC from oncocytic PDTC [16–18]. The concept of tumor progression from PTC and FTC to PDTC can also be observed on the miRNA level where several miRNAs known to be upregulated in thyroid cancer like miR-221 and miR-222 are increasingly deregulated in PDTC as compared with PTC [18].

Long non-coding RNAs (lncRNAs) are other non-protein coding transcripts longer than 200 nucleotides that regulate proliferation and tumor recurrence. Initial evidence shows that lncRNAs may be involved in thyroid tumor progression and that they play a role in *BRAF* regulation and in the MAPK pathway [46].

## Prognosis

Poorly differentiated thyroid carcinomas are aggressive lesions that require appropriate diagnosis, treatment, and follow-up. The relapse-free survival is less than 12 months, the mean survival is 50–60 months, and 44% of patients die of disease [2, 14]. Several factors have been identified to affect patient prognosis. Among them are increased patient age of ≥45 years, large tumor size of ≥5 cm, macroscopically evident extrathyroidal extension at surgery and distant metastases at presentation [33], immunohistochemical markers like insulin-like growth factor mRNA-binding protein 3 (IMP3) and tumor necrosis factor [2].

The mutational landscape of PDTC continues to be deciphered and the identified molecular markers of aggressiveness are *TERT* promoter, *MED12, RBM10, BRAF, HRAS, TP53, ATM, *and* EIF1AX* mutations [24, 32, 55]. Deregulation in the expression of miR-23b and miR-150, both well-known tumor-associated miRNAs, also seem to play a role in PDTC and are suitable for predicting patient outcome [18].

The oncocytic subtype may be associated with an even worse clinical outcome than conventional PDTC, possibly due to a decreased radioiodine uptake that is typical for oncocytic lesions in general [15].

## Treatment

There is no standardized treatment for PDTC to date. However, it is accepted that in general terms a more aggressive approach is needed to treat these tumors as compared with standard low-risk thyroid carcinomas [59]. If possible, a total thyroidectomy including lymph node dissection should be performed. According to one study, total thyroidectomy and removal of residual disease achieved a 5-year regional control of 81% [22]. Radioiodine treatment is only successful in a subset of patients owing to variable levels of iodine uptake, although the exact number of responders is unclear. Nevertheless, given the high mortality rate of the neoplasm and the potential therapeutic benefit, high-dose radioiodine treatment is currently recommended for all PDTC patients by a multidisciplinary group of experts [45]. The same authors recommend external beam radiation for large tumors of >4 cm with stage T3 and T4 and for patients with regional lymph node metastases [45]. However, little has been published on PDTC and external beam radiation and recommendations are solely based on extrapolation from studies of well-differentiated thyroid carcinomas (PTC/FTC) where a treatment benefit could be shown in patients who are at high risk of local recurrences [8]. Chemotherapy is currently not standard of care, although positive effects have rarely been observed in some patients [58]. By contrast, the role of tyrosine kinase inhibitors is evolving and they may be a new and promising approach for treating PDTC in the future. In fact, after the results of the DECISION and the SELECT trial were published, sorafenib and lenvatinib were approved by the U.S. Food and Drug Administration (FDA) for patients with radioiodine-resistant progressive, recurrent, or metastatic disease [10, 25, 47]. However, we still do not know whether a specific molecular signature predicts response to a given tyrosine kinase inhibitor. It is also unknown whether patients should be treated in early or late stages, and the actual benefit in terms of patient survival remains to be seen [52].

## Summary

Poorly differentiated thyroid carcinomas are aggressive lesions that severely impact patients’ life and a correct diagnosis is central for further patient management.

The Turin criteria, the diagnostic algorithm facilitating a diagnosis of PDTC, were established more than 10 years ago and since then their utility has been proven in multiple studies. The former uncertainty among pathologists with these lesions should be resolved and PDTCs are now accepted as a separate entity in the current WHO classification. Some studies of epigenetic changes are available and loss and gains of different chromosomes as well as miRNA deregulation seem to be involved in the development of PDTC. Despite our growing knowledge on molecular alterations in the thyroid, molecular pathology has currently no central role as a diagnostic tool of PDTC, mostly owing to the concept of stepwise thyroid cancer progression. Nevertheless, the role of molecular pathology in conjunction with new tyrosine kinase inhibitors will become increasingly important to ensure adequate treatment in the era of personalized medicine.

## Practical conclusion

- Poorly differentiated thyroid carcinomas are an underdiagnosed entity.
- An increased mitotic index and tumor necrosis in conjunction with a solid trabecular or insular growth pattern as described in the Turin proposal can reliably differentiate between a well-differentiated thyroid carcinoma and a poorly differentiated thyroid carcinoma.
- A poorly differentiated area of only 10% within a thyroid carcinoma will significantly affect patient prognosis.
- Tyrosine kinase inhibitors offer new treatment options in these very difficult to treat tumors.
- Molecular mutational testing will help in decision-making for targeted therapies.
